# Integrating Nature Into Oncology: Enhancing Hematological Malignancies Therapies With Natural Medicine

**DOI:** 10.1002/cam4.71335

**Published:** 2025-11-13

**Authors:** Yaoyao Tian, Baoyi Ni, Dandan Cai, Jia Wang, Jilai Zhou, Mingqian Song, Xi Zhang, Jiakang Jiang

**Affiliations:** ^1^ Heilongjiang University of Chinese Medicine Harbin China; ^2^ The First Affiliated Hospital of Heilongjiang University of Chinese Medicine Harbin China; ^3^ Harbin Vocational and Technical University Harbin China; ^4^ Hongqi Hospital of Mudanjiang Medical University Mudanjiang China; ^5^ Mudanjiang Medical University Mudanjiang China; ^6^ Xuyi County People's Hospital Huai'an China

**Keywords:** combination therapy, hematological malignancies, leukemia, lymphoma, natural medicine

## Abstract

Hematological malignancies are complex clonal disorders that pose a significant threat to human health. Current treatment modalities, including radiotherapy, chemotherapy, immunotherapy, and bone marrow transplantation, have demonstrated efficacy but are often limited by side effects, drug resistance, and suboptimal cure rates. Natural medicines, as key elements of complementary and alternative medicine, exhibit considerable potential in combating hematological cancers. Particularly when used in combination with conventional therapies, they can enhance treatment efficacy and reverse drug resistance. This review comprehensively summarizes the mechanisms of action of bioactive natural compounds, extracts, and derivatives against hematological malignancies, with a focus on their roles in apoptosis induction, proliferation suppression, autophagy regulation, cell cycle arrest, and chemosensitization. We also consolidate evidence from preclinical studies and clinical cases supporting their combined use with standard treatments. By elucidating the therapeutic potential and mechanistic insights of natural medicines, this review aims to provide a foundation for developing more effective and safer treatment strategies, facilitating the translation of natural product‐based therapies into clinical practice for hematological malignancies.

## 1 Introduction

Hematological malignancies are malignant clonal proliferative diseases that arise from the blocked differentiation and development of hematopoietic stem cells. This category encompasses acute and chronic leukemias, lymphomas, multiple myeloma, and other related disorders. Both clinical manifestations and disease outcomes are highly heterogeneous. Globally, these diseases pose a significant threat to human health. According to data from the International Agency for Research on Cancer (IARC) of the World Health Organization [1], approximately 1.31 million new cases were diagnosed worldwide in 2022, accounting for 8.9% of all newly diagnosed cancers. The annual death toll reached 699,300, representing 7.1% of the total cancer‐related deaths. Among these, leukemia is one of the most common types of malignancies in children [2], primarily including acute lymphoblastic leukemia (ALL), acute myeloid leukemia (AML), chronic lymphocytic leukemia/small lymphocytic leukemia (CLL/SLL), and chronic myeloid leukemia (CML). Approximately 486,700 new cases of leukemia are diagnosed annually. Lymphomas, including Hodgkin Lymphoma (HL) and Non‐Hodgkin Lymphoma (NHL), are relatively common among adults, with approximately 635,400 new cases reported annually [3]. These diseases have a relatively high mortality rate, imposing a significant burden on patients and their families. Although the overall incidence of hematologic malignancies is generally lower than that of solid cancers, certain subtypes exhibit high aggressiveness, depending on the disease type and stage. The prognosis of malignant hematologic malignancies has significantly improved with current treatment strategies combining chemotherapy, stem cell transplantation, radiotherapy, and immunotherapy [4]. However, existing drugs have limited efficacy in curing certain hematological malignancies, and they are often associated with high costs, numerous side effects, drug resistance, and low cure rates. Consequently, there is an urgent clinical need for novel therapeutic agents that offer improved safety and efficacy to enhance patient prognosis and quality of life.

**FIGURE 1:**
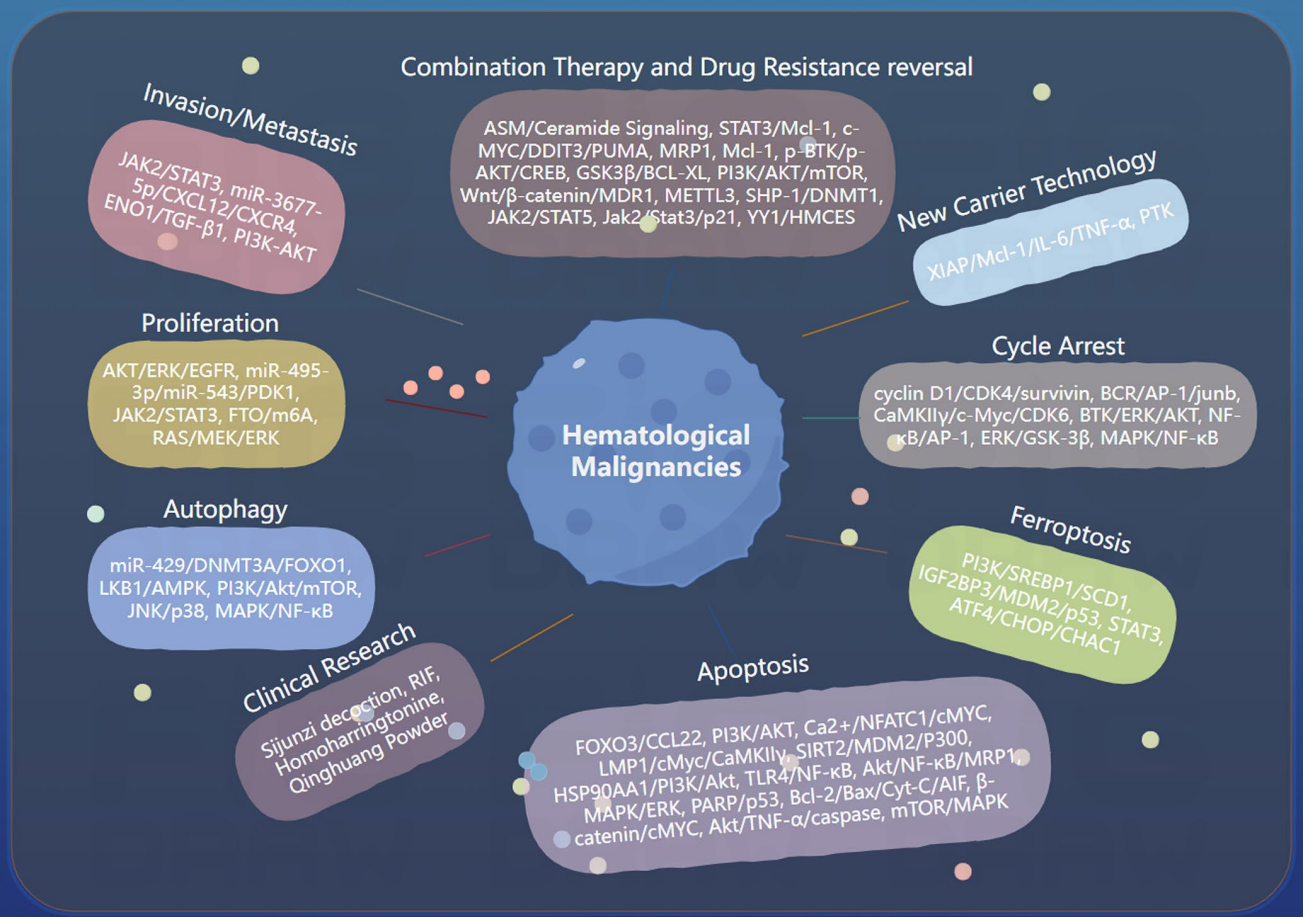
Main mechanisms/pathways of natural medicines against hematologic malignancies.

In recent years, natural medicines have shown considerable potential for the development of anti‐cancer agents [5]. Compared to conventional treatments such as chemotherapy, radiotherapy, and immunotherapy, natural medicines offer benefits including diverse origins, reduced toxicity, and a lower incidence of adverse effects [6]. Many natural medicines exhibit unique chemical structures and biological activities, providing novel insights and directions for anti‐cancer research [7]. This article reviews recent studies on natural medicines with anti‐lymphoma and anti‐leukemia properties, elucidating their respective mechanisms of action in the prevention and treatment of hematologic malignancies. It is anticipated that this review will serve as a useful reference and provide new data to support future drug development and combined treatment strategies. Although natural medicines have shown certain potential in the treatment of hematological malignancies, they still face many challenges, including difficulties in quality control and standardization due to their complex components and diverse mechanisms of action, as well as issues regarding how to better achieve combination applications with existing therapies. These problems require further in‐depth research (Figure 1).

## 2 Inhibit the Proliferation of Cancer Cells

The AKT/ERK/EGFR signaling pathway is a critical intracellular signaling cascade that mediates various physiological processes, including cell proliferation, differentiation, and apoptosis. In cancer cells, aberrant activation of this pathway is closely associated with tumorigenesis and malignant progression. Artesunate (ART), a derivative of artemisinin, has demonstrated significant anti‐cancer activity. In related studies, scholars have investigated the molecular mechanism by which ART inhibits the proliferation of Burkitt's lymphoma (BL) cells through targeting Hsp90. The results indicate that ART binds to the Hsp90 protein and downregulates the expression levels of AKT, ERK, and associated phosphorylated proteins via modulation of the AKT/ERK/EGFR signaling pathway. Further animal experiments have corroborated these findings, confirming that ART suppresses the expression of AKT and ERK proteins in cancer tissues in vivo, thereby effectively inhibiting BL proliferation and leading to a significant reduction in cancer volume and weight. Together, these results suggest that ART holds promise as a novel anticancer agent [8].

Cancer cells predominantly depend on aerobic glycolysis for Adenosine Triphosphate (ATP) production. Within this metabolic pathway, pyruvate dehydrogenase kinase (PDK) acts as a key regulatory enzyme, playing a central role in glucose catabolism. Through its modulation of aerobic glycolysis, PDK contributes significantly to the metabolic reprogramming of cancer cells. As a major subtype of the PDK family, PDK‐1 shows aberrant expression in various cancer tissues and is primarily involved in regulating cell proliferation and migration. Studies have indicated that matrine, an active compound extracted from the traditional Chinese medicine Kushen, can inhibit the Wnt/β‐catenin signaling pathway via the miR‐495‐3p/miR‐543/PDK1 axis. This leads to the downregulation of PDK1 expression, thereby suppressing both proliferation and glycolysis in acute myeloid leukemia (AML) cells and hindering AML progression [9]. In recent years, arsenic trioxide has gained attention as a therapeutic agent for AML. Using network pharmacology approaches, Zeng et al. identified potential synergistic targets to arsenic trioxide and curcumin in AML, including TP53, MAPK3, MAPK1, STAT3, and SRC, and verified them experimentally. Results demonstrated that in the KG1‐a cell line, curcumin enhanced arsenic trioxide‐induced apoptosis and significantly inhibited cell proliferation compared to monotherapy. This was achieved through downregulation of JAK2, STAT3, and Bcl‐2 proteins, alongside up‐regulation of P53, P27, and Bax proteins. In vivo experiments further supported these findings, showing prolonged survival in mice treated with the combination therapy. These results underscore the potential of curcumin and arsenic trioxide as combined treatment strategies for AML, warranting further investigation and validation [10].

Transcription, an essential biological process in all living cells, is particularly critical for cancer cells to sustain the high transcriptional activity required for their rapid proliferation and survival. Transcriptional dysregulation is a hallmark of cancer cells. In hematological malignancies, uncontrolled activity of RNA polymerase II (RNAP II), especially the hyperactivity of its largest subunit, RPB1, is considered crucial for cancer cell survival. As a key oncogenic hub gene, RPB1 plays a decisive role in promoting AML cell proliferation and inhibiting apoptosis. Aberrant DNA damage repair mechanisms significantly contribute to chemotherapy resistance. Upon DNA damage, RPB1 binds to the Williams syndrome transcription factor (WSTF) to form the WSTF‐RPB1 complex, which participates in the DNA damage repair process. Consequently, blocking this pathway has become an effective strategy to reverse chemotherapy resistance. Triptolide, a compound with significant broad‐spectrum anti‐cancer activity, has attracted researchers' attention due to its mechanism of action on AML. Kang et al. revealed the underlying mechanism of triptolide's inhibition of AML through their research. The results indicate that triptolide can inhibit the expression of the WSTF‐RPB1 complex in a time‐ and dose‐dependent manner, significantly suppressing the proliferation of AML cells. These findings provide a strong experimental foundation and highlight the clinical potential of triptolide as a novel therapeutic agent for AML [11]. The fat mass and obesity‐associated protein (FTO) is an mRNA N6‐methyladenosine (m6A) demethylase that functions as an oncogene, promoting leukemic oncogene‐mediated cell transformation and the development of leukemia. Researchers, including Sun K, have conducted an in‐depth investigation into the mechanism of action of Saikosaponin‐d (SsD) in AML. The findings reveal that SsD specifically targets the FTO protein by modulating the FTO/m6A signaling pathway, thereby increasing the level of m6A RNA methylation. This effect effectively suppresses AML cell proliferation and impedes malignant progression both in vitro and in vivo, providing a scientific rationale for SsD as a novel treatment strategy for AML [12].

CML is a malignant disease originating from hematopoietic stem cells, characterized by abnormal cell proliferation and differentiation. Asperuloside (ASP), a compound extracted from plants of the Rubiaceae and Eucommiaceae families, has been revealed by modern pharmacological studies to exhibit various pharmacological activities, including anti‐inflammatory, analgesic, and hypotensive effects. However, the pharmacological role of ASP in CML treatment and its underlying mechanisms remain inadequately elucidated. The RAS/MEK/ERK pathway is frequently aberrantly activated in tumors and plays a critical role in promoting tumorigenesis and progression. Recent studies have indicated that in the K562 cell model of CML, ASP can modulate the RAS/MEK/ERK signaling pathway, inhibit the expression of related genes, and subsequently promote cell apoptosis and differentiation. This effectively suppresses the malignant proliferation of K562 cells, demonstrating significant anti‐CML activity [13]. Furthermore, daphnoretin, a compound extracted from Chinese redbud, has also been shown to inhibit CML cell proliferation by increasing STAT3 phosphorylation in K562 and HEL cells [14].

## 3 Induced Apoptosis of Cancer Cells

Apoptosis, a form of programmed cell death, plays an essential role in maintaining normal physiological homeostasis. Dysregulation of apoptotic processes is recognized as a key factor in the progression of malignant disease. In recent years, plant‐derived natural compounds such as ginsenoside, baicalin, and cinobufagin have demonstrated significant anti‐proliferative effects against hematological malignancies. Induction of apoptosis represents a major way through which these natural medicines exert their anticancer activity. Studies have revealed that such compounds can promote apoptosis in hematologic cancer cells via modulation of specific signaling pathways. These findings not only provide novel insights into therapeutic strategies for blood cancers but also offer promising candidate molecules for future drug development.

Diffuse large B‐cell lymphoma (DLBCL) is one of the most common subtypes of lymphomas in adults and is characterized by considerable heterogeneity in clinical presentation and prognosis. It accounts for approximately 31% to 34% of NHL cases. Although chemotherapy remains the cornerstone of treatment, its ability to substantially improve survival outcomes remains limited [15]. Therefore, exploring novel therapeutic strategies to enhance chemotherapy efficacy and extend patient survival is of great clinical importance. In this context, the growing recognition of the anti‐cancer properties of natural medicines offers a promising direction for DLBCL treatment.

Baicalin, a principal bioactive component of the traditional Chinese medicine Scutellaria baicalensis, demonstrates a range of pharmacological properties. Studies conducted in DLBCL cell lines have revealed its capacity to promote apoptosis. Further mechanistic investigations have demonstrated that baicalin triggers the apoptotic pathway by significantly elevating intracellular Reactive Oxygen Species (ROS) levels [16]. The FOXO3/CCL22 pathway mediates the recruitment of regulatory T cells (Tregs) into the tumor microenvironment by regulating the expression of the chemokine CCL22, thereby promoting tumor immune escape. Specifically, in SNK‐6 and YTS cells, baicalin regulates the FOXO3/CCL22 signaling pathway, leading to the upregulation of FOXO3 protein expression and the downregulation of CCL22 protein expression, thereby promoting cell apoptosis. Experimental results demonstrated a significantly higher apoptosis rate in baicalin‐treated groups compared with controls (SNK‐6: 32.40 ± 0.37 vs. 5.93 ± 0.74; YTS: 36.27 ± 1.06 vs. 5.93 ± 0.74), with the effect showing clear dose‐dependency. Additionally, animal experiments have also confirmed the activity of baicalin in inhibiting cancer growth [17]. Nobiletin has been demonstrated to possess activity that induces apoptosis in DLBCL cells. The PI3K/AKT pathway is frequently altered in cancer, promoting tumor cell survival, proliferation, and drug resistance. Studies have revealed significant interactions between nobiletin and the AKT1, TP53, and CASP3 proteins, enabling it to exert its biological effects through modulation of the PI3K/AKT signaling pathway. The pro‐apoptotic effect of nobiletin in SU‐DHL‐4 cells is mediated via the suppression of survival signals (p‐PI3K, p‐AKT, and BCL‐2), accompanied by the upregulation of key pro‐apoptotic proteins, including caspase3 and BAX [18]. Bufalin (BF), a toxic cardiotonic steroidal compound isolated from the traditional Chinese medicine Chan Su, bears a high similarity in molecular structure to digitalis compounds. Recent studies have unveiled the remarkable anti‐cancer potential of bufalin. The Ca2+/NFATC1/cMYC signaling pathway drives aberrant tumor cell proliferation, metabolic reprogramming, and immune evasion by integrating calcium signals with oncogenic transcriptional programs. Its dysregulated activation is closely associated with progression and therapy resistance in multiple malignancies. Specifically, in DLBCL cells, BF triggers apoptosis by modulating the Ca2+/NFATC1/cMYC signaling pathway, exhibiting both time and dose dependency. Furthermore, in vivo experiments have provided additional evidence that BF can effectively suppress the growth of DLBCL xenograft cancers in a NOD/SCID mouse model, without causing weight loss in the mice during the treatment process [19]. These results highlight baicalin, nobiletin, and BF as viable candidates for future drug development against DLBCL, indicating substantial promise for clinical translation.

BL is a highly aggressive malignancy associated with poor prognosis [20, 21]. Current treatment regimens primarily consist of chemotherapy, radiotherapy, and targeted therapy. However, the heterogeneity of BL and the development of resistance to existing therapies necessitate the exploration of novel treatment strategies. In recent years, advances in understanding apoptotic mechanisms have revealed that certain compounds can induce apoptosis in BL cells via specific signaling pathways. For example, several natural products and their derivatives have shown efficacy in inhibiting BL cell proliferation and promoting apoptosis, offering new perspectives for BL treatment. One study demonstrated that *Celastrus orbiculatus* extract (COE) upregulates the expression of pro‐apoptotic proteins BAX and Cleaved‐Caspase‐3 while downregulating anti‐apoptotic proteins Bcl‐2 and Bcl‐xL in cancer cells. This effect induced apoptosis in mouse cancer models, reduced transplanted cancer volume, and extended survival, providing new experimental evidence for the potential application of COE in BL therapy [22].

Natural killer/T‐cell lymphoma (NKTCL) is an aggressive subtype of NHL, comprising 5%–20% of all NHL cases and showing a strong association with Epstein–Barr virus infection [23]. The pathogenesis of NKTCL is complex and involves dysregulation of multiple signaling pathways. Current evidence suggests that overexpression of the C‐Myc protein is significantly correlated with poor prognosis and aggressive disease progression in NKTCL. Matrine, a major alkaloid derived from the traditional Chinese medicine Kushen, has demonstrated broad biological activities, including inhibition of proliferation and induction of apoptosis in various cancer types. In a study by Gu et al., the molecular mechanism underlying matrine's suppressive effect on NKTCL malignancy was systematically investigated. Results revealed that in the NK92 cell line, matrine promotes apoptosis and restrains malignant progression by modulating the LMP1‐c‐Myc and CaMKIIγ‐c‐Myc signaling axes. Specifically, matrine downregulated Bcl‐2 expression, enhanced proteasome‐mediated degradation of c‐Myc protein, reduced phosphorylation at the c‐Myc Ser62 site, and suppressed the expression of CaMKIIγ, the key regulatory factor [24]. In addition, Cistanche, another traditional Chinese medicinal herb with a long history of use, has garnered increasing interest for its potential anti‐tumor properties. Recent studies have begun to elucidate the anti‐cancer effects of Cistanche phenylethanoid glycosides (CPhGs), particularly their mechanistic actions against NKTCL cells. PTEN modulates the Bax protein to promote tumor cell apoptosis, and its loss of function is strongly associated with the progression of various cancers. The research indicates that CPhGs upregulate P53 protein levels and trigger cellular apoptotic pathways by inhibiting the SIRT2‐MDM2/P300 and PI3K/AKT signaling pathways while activating the PTEN‐Bax cancer suppressor signal. These findings elucidate the mechanism of action of CPhGs in NKTCL cells, providing novel insights and potential drug candidates for NKTCL treatment strategies [25].

CML is a malignant clonal disorder arising from hematopoietic stem cells in the bone marrow, characterized primarily by the uncontrolled proliferation of white blood cells. Currently, tyrosine kinase inhibitors (TKI) such as Imatinib (IM) and Dasatinib constitute the cornerstone of CML treatment. However, the development of drug resistance significantly compromises their long‐term efficacy. Therefore, exploring novel drug combinations and targeted therapeutic strategies has become an essential focus in both basic clinical research. As an emerging research methodology, network pharmacology provides a powerful perspective for elucidating disease mechanisms and drug actions. Using this approach, Yao et al. predicted the active components of Indigo Naturalis and their key target genes in CML treatment, followed by experimental validation. Results revealed that Indirubin is the primary active constituent of Indigo Naturalis, with HSP90AA1 as its direct core target. By inhibiting HSP90AA1 expression and the PI3K/Akt signaling pathway, Indirubin reduces mitochondrial membrane potential and upregulates apoptosis‐related proteins, thereby promoting apoptosis in HL‐60 cells and exerting anti‐CML effects [26]. Similarly, the Ethanol extract of Cauligaena Spatholobi (EECS) was investigated using the same strategy. EECS was shown to induce apoptosis in K562 cells and suppress cancer progression by upregulating Bax and Caspase‐3 and downregulating Bcl‐2 [27]. These findings offer new perspectives for CML treatment and provide a scientific basis for developing novel combination regimens and targeted therapy.

AML is a hematologic malignancy characterized by high heterogeneity and rapid progression. The development of effective treatment strategies and accurate prognostic assessment for AML remains a major focus in hematology research. In this context, natural medicines have attracted growing interest for their therapeutic potential. For instance, active compounds derived from traditional Chinese medicine, including Ferulic Acid (FA), Resveratrol (RE), Platycodin D (PD), Tanshinone IIA (TAIIA), and ART, have been shown to promote apoptosis in AML cells through diverse molecular mechanisms.

Ferulic acid (FA), the primary active component of the Chinese herbal medicine Asafoetida, has been identified as a novel FGFR1 inhibitor, with IC50 values of 3.78 μM and 12.5 μM for FGFR1 and FGFR2, respectively [28]. In the AML U937 cell line, FA upregulates the expression of Bax and Caspase‐3 while downregulating CyclinD1, CyclinE, Bcl‐2, TLR4, and NF‐κB mRNA levels via modulation of the TLR4/NF‐κB signaling pathway, thereby promoting apoptosis [29]. In contrast, RE suppresses Bcl‐2 expression and enhances caspase‐3 cleavage and activation through the Akt/NF‐κB signaling pathway, leading to apoptosis in HL60 and U937 cells. Furthermore, RE inhibits the expression of the multidrug resistance‐associated protein MRP1, reverses chemoresistance, and exhibits synergistic anti‐AML effects with FA [30].

PD, a triterpenoid saponin derived from the roots of *Platycodon grandiflorum* , has been shown to suppress AML progression through modulation of the PI3K/AKT and MAPK/ERK signaling pathways. It upregulates the expression of pro‐apoptotic proteins BAK and BIM, thereby triggering the mitochondrial‐dependent apoptotic pathways [31]. TAIIA, a major active component of *Salvia miltiorrhiza* , was investigated by Song et al. for its effects on THP‐1 cells. The results demonstrated that TAIIA significantly induces apoptosis by enhancing p53, Bax/Bcl‐2, PARP, caspase‐3 activation, and oxidative stress pathways [32]. Furthermore, ART has exhibited notable anti‐cancer activity in AML. Studies by Liu et al. revealed that ART promotes apoptosis in AML cells through increased ROS production and upregulation of the pro‐apoptotic protein Bim. In a C57 mouse leukemia model, ART delayed AML progression and extended survival while maintaining a favorable safety profile [33]. These findings highlight the potential of traditional Chinese medicine‐derived components as novel therapeutic strategies for AML and support their further development as anti‐leukemia agents.

APL, a specific subtype of AML, is a hematologic malignancy driven by the promyelocytic leukemia‐retinoic acid receptor A (PML‐RARA) fusion gene. The development of treatment strategies for APL has been a major research focus. Recent discoveries regarding natural medicines have brought new promise to this field.

Realgar, an arsenic‐containing traditional Chinese medicinal material, provides greater administration convenience in its oral form compared to injection therapy. Li et al. investigated the pro‐apoptotic mechanism of realgar in the APL cell line (NB4), revealing that it induces apoptosis by modulating the Bcl‐2/Bax/Cyt‐C/AIF signaling pathway, thereby altering the expression of key apoptotic proteins [34]. Cinobufagin (CBG), a primary active component of toad venom, has demonstrated efficacy in suppressing APL progression. Studies indicate that CBG regulates the β‐catenin signaling pathway, modulates the expression of Bax, Bcl‐2, cyclin D1, and c‐myc, and induces caspase‐dependent apoptosis in NB4 and NB4‐r1 cells along with PML‐RARA degradation, offering a novel therapeutic strategy for APL patients [35].

20(S)‐Ginsenoside Rh2 (GRh2), a primary active component of ginseng, exhibits a wide range of pharmacological effects, including significant inhibitory actions on various cancers [5]. The Akt/TNF‐α/caspase signaling pathway plays a key role in tumorigenesis, progression, and therapy resistance by regulating apoptosis, inflammatory responses, and survival signals. Studies have shown that GRh2 inhibits APL progression. Specifically, GRh2 modulates the Akt/TNF‐α/caspase pathway, triggering robust ROS generation and mitochondrial impairment. It upregulates caspase3/8/9 and TNF‐α expression, suppresses Akt phosphorylation, and promotes PML‐RARA degradation along with caspase‐dependent apoptosis in NB4 cells [36]. Additionally, honokiol (HNK), a natural bioactive compound, activates the mTOR and MAPK signaling pathways, induces endoplasmic reticulum stress, and facilitates apoptosis, thereby exerting an anti‐APL effect [37]. Collectively, these findings indicate that natural drug‐induced apoptosis pathways represent a promising therapeutic strategy for APL. This approach enhances the understanding of apoptosis‐like cell death mechanisms and offers valuable insights for future clinical research.

## 4 Induced Autophagy of Cancer Cells

Autophagy is a catabolic process responsible for the degradation of cytoplasmic components and organelles within lysosomes. Its role in tumorigenesis and progression has been extensively studied. Consequently, targeted therapeutic strategies modulating autophagy signaling pathways have become a research focus in oncology. Its potential in cancer suppression, harnessing autophagy may represent an effective approach to inhibit cancer development.

DLBCL is the most common subtype of NHL, accounting for almost one‐third of all NHL cases. Palmitic acid (PA), a saturated higher fatty acid widely found in various plants, has demonstrated significant anti‐cancer activity. The miR‐429/DNMT3A/FOXO1 signaling axis promotes tumor malignancy through epigenetic mechanisms, influencing cell proliferation, invasion, and chemoresistance. Studies have revealed that in DLBCL, PA can upregulate the expression levels of MiR‐429 and FOXO1 while downregulating the expression of DNMT3A by modulating the miR‐429/DNMT3A axis. This leads to reduced methylation of FOXO1, thereby promoting autophagy and suppressing lymphoma progression. Furthermore, in vivo experiments confirmed that PA inhibits the growth of transplanted cancers. These findings suggest that palmitic acid represents a potential new therapeutic target and a promising research direction for DLBCL treatment [38].

AML is a malignant proliferative disorder originating from myeloid hematopoietic stem/progenitor cells. It is characterized by the abnormal accumulation of primitive and immature myeloid cells in the bone marrow and peripheral blood. Most patients experience rapid disease progression and face a poor prognosis. Currently, there is no definitive cure for AML. In clinical practice, sequential combination chemotherapy regimens, often integrated with traditional Chinese medicine, are used to stabilize the condition and improve long‐term outcomes. Dihydroartemisinin (DHA), a derivative of the traditional Chinese medicinal herb *Artemisia annua* , exhibits broad pharmacological activities. Studies indicate that in HL‐60 and Kasumi‐1 cell lines, DHA upregulates the expression of autophagy‐related proteins, enhances autophagy flux, and induces oxidative stress, thereby inhibiting cell viability and impeding AML progression [39]. Acetylshikonin (ASK), a natural naphthoquinone derivative derived from *Lithospermum erythrorhizon* , has demonstrated significant antibacterial, anti‐inflammatory, and anti‐cancer activities. Investigating the molecular mechanism of ASK‐induced autophagy in AML cells, Wu et al. reported that in HL‐60 cells, ASK promotes the conversion of microtubule‐associated protein light chain 3B (LC3B) from type I to type II via modulation of the LKB1/AMPK and PI3K/Akt/mTOR signaling pathways. This leads to a decreased p62 expression, facilitating autophagosome formation and ultimately inducing autophagy [40]. These findings reveal the potential of both ASK and DHA as therapeutic agents for AML and suggest promising prospects for their clinical application.

APL is associated with considerable early mortality and ranks among the most severe forms of leukemia. Studies using the APL‐derived NB4 cell model have shown that shikonin, compared to controls, significantly suppresses the phosphorylation of PI3K, Akt, and mTOR by modulating the PI3K/Akt/mTOR signaling pathway. This, inhibition subsequently promotes autophagy and demonstrates notable anti‐cancer activity [41].

Resistance to TKIs poses a major challenge in the treatment of CML. Hinokiflavone (HF), a natural biflavonoid derived from the Selaginella species, has shown diverse pharmacological activities. It has garnered considerable attention owing to its low toxicity and anti‐cancer properties. The JNK/p38 and MAPK/NF‐κB pathways regulate inflammatory responses, cellular stress, and survival in cancer, and their sustained activation promotes tumorigenesis, progression, and drug resistance. Qin et al. investigated the anti‐cancer mechanisms of HF in CML cells. The results demonstrated that HF upregulates LC3‐II expression and promotes p62 degradation by modulating the JNK/p38 MAPK/NF‐κB signaling pathway, thereby inducing autophagy and suppressing cell viability. These findings suggest that HF may serve as a novel and effective therapeutic agent for CML [42].

## 5 Inhibition of Cancer Cell Invasion/Metastasis Process

The invasive and migratory properties of cancer cells are hallmark manifestations of their malignant behavior. These characteristics are pivotal to cancer progression and poor treatment outcomes, profoundly impacting patient prognosis. In the pursuit of more effective therapeutic strategies, researchers have increasingly focused on natural medicines. The elucidation of their pharmacological effects has thus introduced novel concepts and opportunities for cancer treatment.

The JAK2/STAT3 signaling pathway is aberrantly activated in tumors, promoting cell proliferation, suppressing apoptosis, and inducing immune microenvironment dysregulation. Targeted inhibition of this pathway has become an important strategy in cancer therapy. In a study on DLBCL, the mechanism of DHA action was investigated in OCI‐Ly7 cells. Results showed that DHA effectively suppresses cancer stem cell‐like properties and significantly inhibits invasion and migration by regulating the JAK2/STAT3 signaling pathway and reducing the phosphorylation of JAK2 and STAT3. DHA also exhibited potent cytotoxicity and anticancer effects. These findings provide supportive evidence for the potential application of DHA in lymphoma treatment [43].

AML, classified as the M5 subtype of AML under the FAB system, is characterized by a high frequency of extramedullary infiltration (EMI) and an unfavorable prognosis. Ginseng, a traditional Chinese medicine, exhibits diverse pharmacological activities. Ginsenoside Rk3 (GRk3), one of its main active components, was investigated by Ma et al. for its mechanism against EMI in monocytic leukemia cells (SH‐1). The results demonstrate that GRk3 effectively modulates the miR‐3677‐5p/CXCL12 axis, leading to downregulation of CXCL12 and CXCR4, upregulation of miR‐3677‐5p, and subsequent regulation of MMP2 and TIMP2 expression, thereby significantly suppressing EMI. This study enhances the understanding of ginsenoside's pharmacological properties and provides compelling evidence for its therapeutic potential [44].

Research indicates that approximately 90% of BL patients exhibit abnormal karyotypes in their cancer cells, frequently involving translocations of chromosomes 8 and 14, as well as rearrangements of the c‐MYC oncogene. These genetic alterations are thought to contribute to disease pathogenesis. Enolase 1 (ENO1), a key glycolytic enzyme, plays an important role in multiple pathological processes, including cancer development. The overexpression of ENO1 is closely related to the proliferation, migration, and invasion abilities of cancer cells. Wang and other researchers have discovered a novel ENO1 inhibitor called Ciwujianoside E (C‐06). This inhibitor reduces the recruitment of plasminogen (PLG), the generation of plasmin, and the activation of TGF‐β1 by downregulating the expression of ENO1. Furthermore, C‐06 disrupts the interaction between PLG and ENO1, inhibits the PI3K‐AKT signaling pathway and the process of Epithelial‐Mesenchymal Transition (EMT), thereby attenuating the invasiveness of BL cells. These findings suggest that C‐06 may represent a potential therapeutic agent for BL with promising clinical translational prospects [45].

## 6 Regulation of Cancer Cell Cycle Arrest

Cell cycle arrest refers to the cessation of progression through a specific phase of the cell cycle, typically induced by DNA damage or cellular stress. In cancer cells, dysregulation of the cell cycle can result in uncontrolled proliferation, thereby driving cancer initiation and progression. Therefore, targeted pharmacological intervention in the cell cycle can effectively arrest it at a specific stage, inhibiting the proliferation of cancer cells. For instance, certain natural medicinal components such as polyphyllin, ginsenoside, and artesunate have been demonstrated to induce cell cycle arrest. The identification of these natural agents provides new insights and potential therapeutic strategies for the treatment of hematological malignancies.

Despite advances in the treatment of DLBCL, patient survival remains suboptimal, underscoring the need for novel therapeutic strategies. Polyphyllin (PP), a primary active constituent of the traditional Chinese medicinal herb Chonglou, and its subtype PP VII, have attracted attention for their potential role in DLBCL. Studies demonstrate that PP VII suppresses malignant proliferation in U2932 and SUDHL‐4 cell lines by inducing cell cycle arrest at the G0/G1 phase, significantly reducing the proportion of cells in the G2/M phase, and downregulating the expression of cyclin D1, CDK4, CDK6, and survivin proteins. These findings highlight the potential value of PP VII in anti‐lymphoma therapy [46]. In a related study, Lai et al. investigated the therapeutic potential of Dihydrocelastrol (DHCE), a derivative of celastrol, in DLBCL. Their findings indicated that DHCE induces G0/G1 phase arrest via modulation of the BCR/AP‐1/JunB signaling pathway. Moreover, DHCE exhibits a synergistic effect with Doxorubicin (DOX), significantly enhancing anti‐lymphoma efficacy [47]. Matrine, an alkaloid derived from Radix Sophorae Flavescentis, displays diverse pharmacological activities. Research has shown that in DLBCL cells, matrine shortens the half‐life of c‐Myc protein (from 30.4 and 69.4 min to 16.6 and 15.9 min, respectively) by regulating the CaMKIIγ/c‐Myc/CDK6 axis. Phosphorylation of CaMKIIγ, a critical kinase for c‐Myc stability, is also downregulated. Additionally, matrine induces dose‐dependent G0/G1 phase arrest, effectively inhibiting the malignant progression of SU‐DHL‐16 and OCI‐LY3 cells [48].

Myricetin (MYR), a polyhydroxy flavonoid originally identified in waxberry and commonly present in various medicinal plants, has garnered research interest for its bioactivity. Bruton's tyrosine kinase (BTK), a key kinase in the B‐cell receptor signaling pathway, is widely expressed in several hematologic malignancies and regulates B‐cell proliferation, differentiation, and apoptosis, making it an attractive therapeutic target. Song et al. investigated the mechanism of MYR targeting BTK and demonstrated that MYR binds key residues (Ala478, Leu408, Thr474) within the BTK, active site, inhibits tyrosine 223 autophosphorylation, and blocks downstream BTK/ERK and BTK/AKT signaling, functioning as a natural BTK inhibitor. Furthermore, MYR induces cell cycle arrest in TMD‐8 cells by modulating cyclin B1/D1 expression. In vivo studies confirmed that oral administration of MYR significantly suppresses tumor growth without notable toxicity. These results support MYR, as an effective natural BTK inhibitor and a promising treatment option for DLBCL [49].

Primary effusion lymphoma (PEL), a condition associated with Kaposi's sarcoma‐associated herpesvirus (KSHV) infection, often carries a poor prognosis. Ishikawa et al. investigated the effects of ART on PEL cell lines and found that ART downregulates the expression of Cyclin D1/D2, CDK2/6, and c‐Myc via the NF‐κB/AP‐1 signaling pathway, inducing G1 phase cell cycle arrest. ART increased ROS production and promoted DNA damage, thereby slowing PEL progression in a mouse model. These results support the potential of ART as an effective agent against PEL [50]. In a separate study, Jiang et al. explored the mechanism of ginsenoside Rd. (GRd) in AML. Their work revealed that GRd modulates the ERK/GSK‐3β pathway, resulting in downregulation of WT1 and upregulation of GATA‐1 expression. GRd also induced G0/G1 phase cell cycle arrest and, in animal models, significantly reduced cancer weight and volume. These findings indicate that GRd may effectively inhibit AML progression [51].

The development of resistance to TKI represents a major obstacle in the treatment of CML. Qin et al. investigated the anticancer activity of HF in K562 cells and demonstrated that HF induces G_2_/M phase cell cycle arrest by modulating, the MAPK/NF‐κB signaling pathway, up‐regulating p21 expression, and downregulating Cdc2 levels, thereby suppressing cell survival [42]. Sinomenine, a key active compound derived from the traditional Chinese medicine Qingfengteng, has been widely used for rheumatic diseases. Meanwhile, 8‐Bis(benzylthio)octanoic acid (CPI‐613), an orphan drug approved for specific resistant malignancies, has also gained clinical interest. Gao et al. synthesized a novel compound (S‐CPI) by chemically linking sinomenine and CPI‐613, and evaluated its therapeutic potential in CML. Results showed that S‐CPI exhibited significant cytotoxicity in K562 cells (IC50 of 2.45 μM) and induced G_1_ phase cell cycle arrest, effectively inhibiting CML progression [52]. These findings highlight the potential of HF and S‐CPI as novel candidate agents for CML therapy. In addition, Polyphyllin VII (PPVII) has shown broad anti‐cancer activity. Lin et al. explored its mechanism in human erythroleukemia (HEL) cells and revealed that PPVII induces S phase arrest through the mitochondrial pathway, accompanied by loss of mitochondrial membrane potential, thereby exerting potent anti‐leukemic effects. These results support PPVII as a promising alternative therapeutic candidate for leukemia treatment [53].

## 7 Combination Therapy and Drug Resistance Reversal

Resistance to chemotherapeutic agents represents a significant obstacle in oncology, often leading to the failure of initially effective treatments. In hematological malignancies, the emergence of drug resistance frequently results in diminished or lost responsiveness to conventional therapies. To address this challenge, multiple strategies are being actively investigated. Combination therapy, which leverages synergistic mechanisms among different agents, such as integrating natural medicines with mainstream antitumor regimens, can enhance efficacy and delay the onset of resistance. Furthermore, the development and application of novel carrier technologies, such as nano‐drug delivery systems, provide new avenues to improve the stability and targeting of drugs in the body, thereby helping to overcome drug resistance. With a deeper understanding of cancer drug resistance mechanisms and the continuous emergence of novel drugs and therapeutic strategies, we can synergistically enhance treatment efficacy, reduce toxic and side effects, improve survival rates, and prolong survival periods. These developments will provide more effective therapeutic alternatives for patients with hematological malignancies and contribute to overcoming the challenge of treatment resistance.

PEL carries a median survival of < 6 months, reflecting an extremely poor prognosis. Scholars have conducted in‐depth research on the mechanism of DHA in the treatment of PEL. The results show that in vitro experiments, DHA can synergize with DOX, enhancing its induction of caspase‐dependent apoptosis, demonstrating a synergistic effect. In vivo experiments, DHA treatment significantly reduces cancer volume and inhibits cell proliferation. These collective findings suggest the possibility of DHA as a potentially effective candidate drug for PEL therapy [54]. Resistance to rituximab (RTX) poses a significant challenge in the treatment of DLBCL. Beta‐sitosterol (β‐ST), a plant sterol widely distributed in nature, has demonstrated anti‐cancer properties in various solid malignancies. He et al. explored the mechanism of β‐ST in DLBCL and found that it modulates sphingolipid metabolism, promotes acid sphingomyelinase (ASM) to the plasma membrane, and regulates the ASM/ceramide signaling pathway, thereby enhancing the therapeutic effect of RTX on DLBCL. The ability of β‐ST to reverse drug resistance offers a novel direction for developing innovative DLBCL treatment strategies [55].

The emergence of targeted therapies represents a paradigm shift in anti‐cancer treatment from traditional broad‐spectrum cytotoxicity toward precision targeting, thereby minimizing damage to normal tissues. However, in cases of strong treatment resistance and persistence, such as DLBCL, the efficacy of relying solely on targeted therapies remains unsatisfactory. Chen and other researchers conducted an in‐depth exploration of the therapeutic effects of combining ART and sorafenib (SOR) in DLBCL SU‐DHL4 cells. The study results indicate that ART significantly enhances SOR‐induced apoptosis and ferroptosis effects by inhibiting the STAT3 signaling pathway. Additionally, it downregulates the expression levels of glutathione peroxidase 4 and myeloid cell leukemia‐1 (Mcl‐1). Furthermore, the combined treatment regimen inhibits CD31 expression in animal models, thereby suppressing angiogenesis. The synergistic application of ART and SOR offers a novel therapeutic avenue and compelling experimental support for the treatment of refractory NHL [56].

Cutaneous T‐cell lymphoma (CTCL) is a rare type of NHL characterized by the malignant proliferation of T‐cell lymphocytes. Chidamide, a histone deacetylase inhibitor, is an established therapeutic agent for CTCL. Matrine, a major active alkaloid derived from the traditional Chinese medicine kushen, exhibits diverse pharmacological effects. He and their research team conducted an in‐depth study on the impact of the combined application of Chidamide and matrine on the treatment effect of CTCL. The results showed that compared to Chidamide monotherapy, the addition of matrine could upregulate the expression level of caspase‐3, thereby enhancing the apoptosis induced by Chidamide. Furthermore, in vivo experiments further confirmed the superiority of the combined treatment regimen in pharmacodynamics, demonstrating more significant anti‐cancer activity. These findings support matrine as a potential adjuvant to chidamide in the management of CTCL [57].

Homoharringtonine (HHT), a plant‐derived alkaloid extracted from the Cephalotaxus genus, exhibits anti‐cancer activity. Azacitidine (AZA), a demethylating agent, is a commonly used drug for the treatment of AML. Through experimental studies, Li and their research team revealed that in the treatment of AML cells U937 and MV4‐11, compared to the use of AZA alone, HHT can trigger cell apoptosis and activate the integrated stress response by modulating the c‐MYC/DDIT3/PUMA signaling pathway, thereby inhibiting cell proliferation. The combined regimen of HHT and AZA exhibits synergistic anti‐AML activity [58]. Cytarabine (ara‐C) is a conventional chemotherapeutic agent for AML, but its long‐term use often leads to drug resistance, necessitating new combination strategies to improve outcomes. Ginsenoside K (GK), as the main metabolite of ginsenoside, exhibits significant anticancer activity. Studies have indicated that the combined application of GK and ara‐C can enhance the DNA damaging effects on AML cells, increase the cytotoxicity of ara‐C, promote cell apoptosis and cycle arrest, reduce chemotherapy resistance caused by ara‐C, and thus optimize the therapeutic effect of ara‐C [59]. Multidrug resistance‐associated protein 1 (MRP1), a drug‐efflux transmembrane transporter, contributes significantly to chemotherapy resistance in AML. Research has found that RE significantly downregulates MRP1 expression, gradually reversing chemotherapy resistance in AML‐resistant cell lines HL60 and U937, and exerting anticancer effects [30].

FMS‐like tyrosine kinase 3 (FLT3) plays a critical role in regulating cell proliferation and differentiation. Approximately 30% of acute myeloid leukemia (AML) patients harbor FLT3 mutations, which are associated with poor prognosis. UBE2L6, a ubiquitin‐conjugating enzyme, plays a crucial role in the E6/E6‐AP‐mediated ubiquitination of p53/TP53, regulating cellular responses to stress and DNA damage. Additionally, UBE2L6 is essential in promoting the ubiquitination of FLT3 and its subsequent proteasomal degradation, making it a key protein in regulating FLT3 activity. While gilteritinib is an effective FLT3 inhibitor, its efficacy remains limited. Cai et al. investigated the mechanism of action of combining HHT with gilteritinib in FLT3‐ITD mutated AML cell lines. Their results demonstrate that HHT upregulates UBE2L6 expression, leading to Mcl‐1 degradation, reduced mitochondrial membrane potential, and enhanced apoptosis. The synergistic effect of HHT and gilteritinib significantly enhances the therapeutic effect, providing new treatment strategies and clinical options for AML patients [60]. Berbamine (BBM), an alkaloid drug derived from Chinese herbal medicine Berberis, has been widely used in clinical practice for a long time. Its main pharmacological effects include anti‐inflammatory, increasing white blood cell count, and anti‐cancer activities. Cui et al. explored the mechanism of BBM combined with ibrutinib (IBR) in AML treatment. Results showed that, compared to IBR alone, the combination significantly enhances apoptosis‐related proteins expression through the p‐BTK/p‐AKT/CREB and GSK3β/BCL‐XL signaling pathways, potentiating IBR‐induced apoptosis and effectively suppressing cell proliferation [61].

ALL represents one of the most frequent malignancies of the circulatory system. Dexamethasone (DEX), a glucocorticoid receptor ligand, serves as a first‐line agent in ALL chemotherapy regimens. However, prolonged or high‐dose DEX administration is associated with adverse effects such as osteoporosis and hypertension, and may also lead to glucocorticoid receptor downregulation and subsequent drug resistance. Therefore, it is crucial to investigate new strategies that can reverse chemotherapy resistance and restore the effectiveness of treatment. Studies have indicated that the combination of Andrographolide (AND) and DEX can enhance the sensitivity of CEM‐C1 cells to DEX by modulating the autophagy‐dependent PI3K/AKT/mTOR signaling pathway. This combination therapy downregulates the expression of LC3 and the autophagy‐related gene Beclin1, synergistically improving DEX's anti‐cancer effects. These findings offer new perspectives and potential clinical approaches for ALL therapy [62]. Additionally, the FA‐2‐b‐β extract derived from Agaricus blazei has been shown to reverse the drug resistance mechanism in T‐ALL cells. It was found that FA‐2‐b‐β modulates the Wnt/β‐catenin pathway, suppresses the expression of drug resistance‐related proteins (including ABCB1, ABCG2, CTNNB, and MYC), reduces the mitochondrial membrane potential of cells, and downregulates the expression of the membrane surface protein MDR1. These effects collectively reverse DEX resistance in CCRF‐CEM and CEM/C1 cells, effectively overcoming the multidrug resistance phenotype [63].

The discovery of lactylation modification has revealed a novel connection between cellular metabolism and epigenetic regulation. As a product of metabolic activity, lactylation plays a crucial role in modulating intracellular metabolism and functional states. In cancer cells, lactylation can modulate metabolic adaptation and invasive capabilities, thereby promoting cancer growth and metastasis. This insight not only elucidates a non‐canonical function of lactate in metabolic and epigenetic regulation but also offers new perspectives and approaches for developing innovative therapeutic strategies. Studies have demonstrated that METTL3 expression is significantly upregulated in ATRA‐resistant APL cells. METTL3 is regulated by lactylation modification, and its elevated expression contributes to ATRA resistance. GRh2 has been shown to counteract this resistance mechanism by suppressing lactylated METTL3, suggesting that GRh2 may serve as a potential inhibitor of lactylated METTL3 to ameliorate ATRA sensitivity in APL [64].

Substantial progress has been achieved in recent years in the treatment of chronic myeloid leukemia (CML), a classic myeloproliferative neoplasm, particularly in the selection and application of tyrosine kinase inhibitors (TKIs) as first‐line therapy. These agents effectively reduce the population of malignant leukemic cells by inhibiting the abnormally active tyrosine kinase encoded by the BCR‐ABL fusion gene. As a first‐generation TKI, IM serves as a cornerstone drug for CML treatment. It exhibits good efficacy and relatively low toxicity, making it suitable for most newly diagnosed CML patients. Nevertheless, long‐term IM use is often associated with the emergence of drug resistance or intolerance, which can significantly compromise clinical outcomes.

Src homology 2 domain‐containing protein tyrosine phosphatase 1 (SHP‐1) functions as a negative regulatory in cellular signaling systems, modulating signal transduction through catalytic dephosphorylation to suppress aberrant pathway activation. Evidence indicates that the SHP‐1 gene is generally unmethylated in healthy individuals, whereas its methylation is associated with IM resistance in CML. Baicalein has been shown to interfere with DNA methyltransferase 1 (DNMT1) expression, leading to demethylation in the SHP‐1 promoter region and subsequent reactivation of SHP‐1. This reactivation leads to the inactivation of the JAK2/STAT5 signaling pathway, reversing IM resistance induced by the bone marrow microenvironment, and inhibiting the malignant progression of CML CD34+ cells [65]. MYR, a naturally derived flavonoid, exhibits multiple biological activities. Recent studies demonstrate that MYR significantly inhibits IM‐resistant CML CD34+ stem/progenitor cells. Specifically, MYR reduces phosphorylation of eIF4E and Akt, and downregulates expression of c‐Myc and Cyclin D1 proteins [66].

Cancer cell senescence describes a state of permanent cell cycle arrest induced in malignant cells by specific stimuli. The induction of senescence represents a promising therapeutic approach, as it can effectively suppress tumor growth and dissemination. Icaritin (ICA), a prenyl flavonoid derived from Epimedium plants, is a traditional Chinese herbal medicine. Studies have demonstrated that ICA significantly inhibits the growth of CML cells. Specifically, ICA regulates the Jak2/Stat3/p21 signaling pathway, increases the accumulation of reactive oxygen species (ROS), and induces senescence in IM‐resistant cells, thereby suppressing the malignant progression of CML [67]. These findings highlight the potential of ICA, along with baicalein and MYR, to reverse chemoresistance, positioning them as novel candidates for the treatment of CML.

In the current therapeutic landscape of CML, overcoming the BCR‐ABL T315I mutation remains a major clinical challenge. HHT, an FDA‐approved natural product, has demonstrated potent anti‐leukemia activity. Han et al. explored the mechanism underlying HHT's efficacy in CML and observed significant downregulation of mitochondrial complex I (MCI) protein expression following HHT treatment. Given the essential role of MCI in oxidative phosphorylation (OXPHOS), this finding indicates severe disruption of OXPHOS, resulting in reduced intracellular ATP levels. This implies that HHT effectively inhibits the proliferation of T315I mutation‐resistant cells by suppressing MCI activity [68]. Additionally, celastrol has been shown to induce apoptosis in BCR‐ABL‐related T315I mutant drug‐resistant cells. Studies have also found that YY1 (Yin and Yang 1 Protein) plays a promoting role in the occurrence and development of various cancers, while HMCES is a protein related to DNA repair. Further research indicates that celastrol induces the accumulation of DNA damage by targeting YY1 and HMCES, ultimately leading to the apoptosis of drug‐resistant K562 cells [69]. This study elucidates a previously unrecognized mechanism of HHT and celastrol, and proposes a novel therapeutic strategy to overcome BCR‐ABL T315I mutation‐mediated resistance.

## 8 Application of New Carrier Technology

The isolation of biologically active compounds from natural medicinal sources for the development of novel preventive and therapeutic agents represents a significant and innovative direction in contemporary pharmaceutical research. However, the diversity and complex pharmacological properties of these compounds often lead to challenges such as poor solubility, limited stability, and low oral absorption, which remain incompletely resolved. These issues frequently result in low bioavailability, posing a major obstacle to achieving the desired therapeutic outcomes. Currently, the development of novel drug delivery systems and formulation technologies, such as nano‐delivery systems and DNA chimeric techniques, is gradually demonstrating its potential to address these challenges and positively impact the improvement of the bioavailability of active ingredients derived from natural medicines.

Currently, conventional treatment strategies for lymphoma face certain limitations, underscoring the urgency for developing novel treatment approaches. Realgar, a traditional Chinese herbal medicine with a long history of application, is gradually being recognized for its value in modern medicine. Researchers, including Ran W, have employed nanotechnology to nano‐process realgar particles, significantly enhancing their physicochemical properties and bioavailability. Experimental results demonstrate that these nanoparticles significantly enhance T‐cell‐mediated cytotoxicity, boost immune cell activity, and effectively induce apoptosis of lymphoma cells. Additionally, these nanoparticles inhibit cell proliferation and angiogenesis, restricting the nutrient and blood supply to lymphoma cells, thereby exhibiting significant efficacy in anti‐cancer therapy [70]. Curcumin has demonstrated inhibitory effects on HL cell growth in vitro, yet its unfavorable pharmacokinetic profile necessitates novel delivery systems for in vivo application. Guorgui et al. developed curcumin‐loaded solid lipid nanoparticles (SLN‐curc) and d‐α‐tocopherol polyethylene glycol succinate nanoparticles (TPGS‐curc) loaded with curcumin to evaluate its efficacy in mice. The results demonstrated that the use of SLN and TPGS enhanced the plasma concentration of curcumin in mice. Compared with controls, the different nanoparticle systems reduced HL progression in vivo (curcumin 35.8%, SLN 50.5%, TPGS 43.0%). Furthermore, curcumin downregulated the expression of XIAP, Mcl‐1, IL‐6, and TNF‐α, synergistically potentiated the anticancer effects of bleomycin and doxorubicin, inhibited cell proliferation, and promoted apoptosis. These findings suggest that curcumin, particularly in nanoformulated delivery systems, represents a promising therapeutic adjuvant worthy of further investigation in HL treatment regimens [71].

The discovery of BCR‐ABL1 TKIs has revolutionized the treatment of CML, with IM serving as a prototypical agent. However, long‐term use of TKIs can lead to drug resistance, severely affecting treatment efficacy and triggering disease relapse. Wang et al. developed a nano‐crystal co‐delivery system combining realgar with IM (As4S4/IMA), aiming to evaluate its synergistic therapeutic effect on CML and the role in reversing drug resistance. The results indicate that As4S4 can degrade the BCR‐ABL1 protein, while IM inhibits protein tyrosine kinase (PTK) activity. Through a folate‐modified nanosystem, As4S4/IMA achieves precise cancer suppression, demonstrating significant therapeutic effects in both in vitro and in vivo experiments. This study offers a novel strategy and effective platform for precision anti‐cancer therapy, underscoring the promise of natural medicines in cancer treatment [72].

DNA intercalators constitute a class of aromatic small molecules that target DNA, through insertion between base pairs. Leveraging a drug–DNA interaction‐based carrier technology, complexes incorporating DNA intercalators exhibit dual targeting anti‐cancer mechanisms that combine the advantages of intercalation and metal‐based drug activity, enabling more efficient and precise cancer treatment. RoyMahapatra D and colleagues developed an ART complex (Artesunate‐Naphthalimide Hybrid Dual Drug, ANHDD) incorporating a naphthalimide intercalating group. Their findings indicate that ANHDD reduces the viability of DLBCL cells, induces cell apoptosis, and inhibits cancer progression in balb/C mice [73].

## 9 Clinical Research

DLBCL often exhibits a suboptimal response to conventional drug therapies, leading to poor long‐term survival outcomes. This study reports the case of an elderly male patient with DLBCL, harboring mutations in both c‐MYC and BCL2. Initially treated with the DA‐EPOCH‐R regimen, the patient later switched to traditional Chinese medicine (Sijunzi decoction) due to tolerability issues, which he continued for 5 years. At the eighth year after post‐diagnosis, follow‐up evaluations indicate no disease progression, absence of adverse drug reactions, in the patient, and a preserved quality of life. This case provides supportive evidence for the potential role of natural medicine in the long‐term management of DLBCL [74].

**FIGURE 2:**
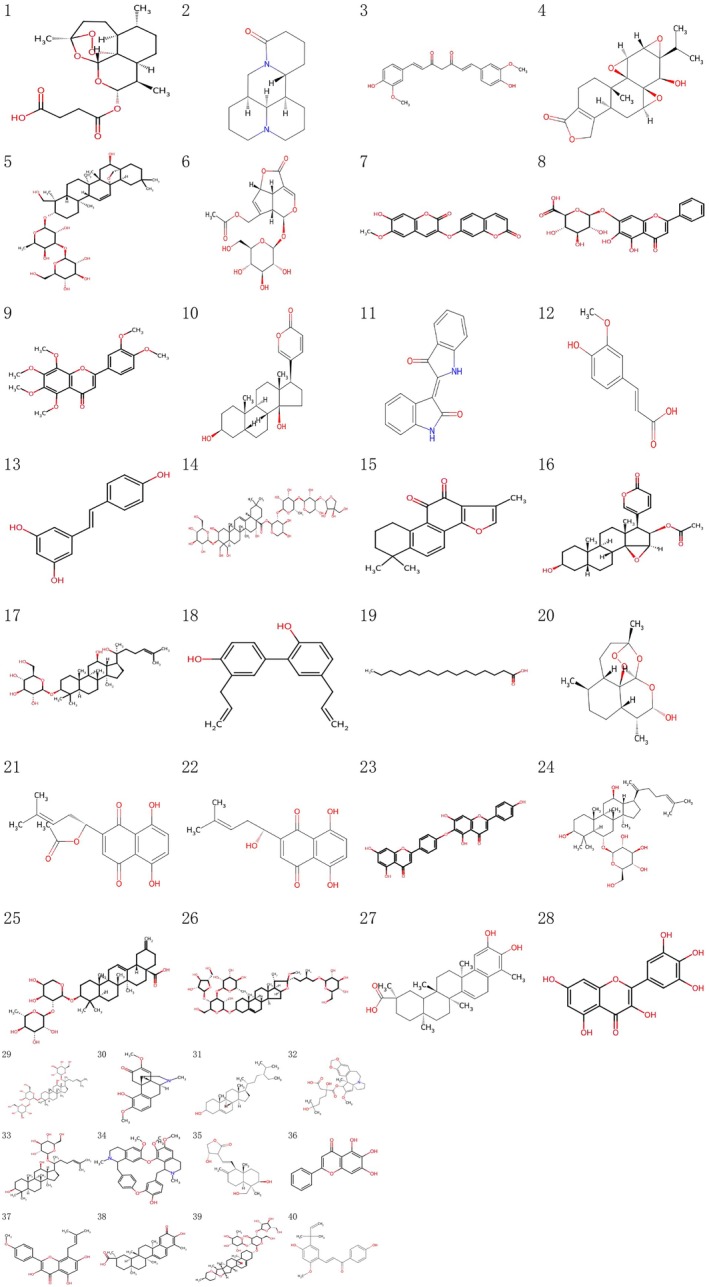
Molecular structural formula of the main active ingredients of natural medicines against hematological malignancies. (1) Artesunate, (2) Matrine, (3) Curcumin, (4) Triptolide, (5) Saikosaponin D, (6) Asperuloside, (7) Daphnoretin, (8) Baicalin, (9) Nobiletin, (10) Bufallin, (11) Indirubin, (12) Ferulic acid, (13) Resveratrol, (14) Platycodin, D (15) Tanshinone IIA, (16) Cinobufagin, (17) 20(S)‐Ginsenoside Rh2, (18) Honokiol, (19) Palmitic acid, (20) Dihydroartemisinin, (21) Acetylshikonin, (22) Shikonin, (23) Hinokiflavone, (24) Ginsenoside Rk3, (25) Ciwujianoside E, (26) Polyphyllin VII, (27) Dihydrocelastrol, (28) Myricetin, (29) Ginsenoside Rd., (30) Sinomenine, (31) β‐Sitosterol, (32) Homoharringtonine, (33) Ginsenoside K, (34) Berbamine, (35) Andrographolide, (36) Baicalein, (37) Icaritin, (38) Celastrol, (39) Polyphyllin I, and (40) Licochalcone A (Image credit: National Library of Medicine—National Center for Biotechnology Information [https://pubchem.ncbi.nlm.nih.gov/] and ChemSpider Search and share chemistry [http://www.chemspider.com/]).

APL achieves a favorable cure rate under the combined treatment of ATRA and ATO. Currently, ATO and ATRA constitute the standard induction chemotherapy for non‐high‐risk APL. In addition, the traditional Chinese medicinal formula Realgar‐Indigo Naturalis Formula (RIF) has demonstrated notable efficacy, with the advantage of oral administration offering greater convenience compared to intravenous ATO (Figure 2). Results from an RCT study on the combination of RIF and ATRA indicate that the plasma arsenic content in patients receiving ATO injection therapy is several times higher than that in patients taking oral RIF, which may have potential adverse effects on patients. Meanwhile, oral RIF significantly reduces the total hospital stay and overall medical expenses for patients, showcasing important economic value [75]. A meta‐analysis involving 775 APL patients comprehensively evaluated the safety and efficacy of RIF. The results revealed that, compared to the ATO control group, the RIF group had a significantly lower incidence of adverse events and markedly lower expenses during the maintenance treatment phase. Mechanistic studies further indicated that RIF promotes APL cells' apoptosis and suppresses disease progression by modulating the expression of key target proteins such as ACHE, NCOA2, and RXRA [76].

APL occurs predominantly in adults; its incidence in pediatric patients warrants clinical attention. A clinical trial involving 19 pediatric patients was conducted to evaluate the plasma arsenic concentration and drug toxicity of RIF and ATO. Results indicated that by Day 7, the plasma arsenic levels had reached steady state in both groups (0.54 μmol/L in the RIF group and 0.63 μmol/L in the ATO group), with similar mean peak‐to‐trough concentrations (0.54 μmol/L in the RIF group and 0.51 μmol/L in the ATO group) [77]. In a separate randomized, multicenter clinical trial including 92 pediatricAPL patients, the safety and toxicity of RIF were further evaluated. After 3 years of follow‐up, the RIF group showed a significantly shorter hospitalization duration compared to the ATO group, with no significant differences in safety profiles or incidence of adverse events. These findings collectively suggest that oral RIF and intravenous ATO exhibit comparable safety and efficacy in pediatric APL treatment. Moreover, RIF offers the additional advantages of reduced hospital stay and lower economic burden, providing valuable insights for clinical decision‐making [78].

While HHT is an established agent in adult AML therapy, its efficacy and safety in pediatric AML have not been fully validated. A multicenter RCT included 1258 patients evaluated HHT‐containing induction regimens, demonstrating superior outcomes compared to etoposide‐based therapy(CR: 79.9% vs. 73.9%; 3‐year OS: 69.2% vs. 62.8%; 3‐year EFS: 61.1% vs. 53.4%). These results highlight the potential value of HHT in pediatric AML, suggesting its promise as an effective treatment strategy [79]. In a retrospective analysis of 80 elderly acute myeloid leukemia (eAML) patients, arsenic‐containing Qinghuang Powder (QHP) was compared with low‐intensity chemotherapy (LIC). The results indicated no significant difference in overall survival rates between the two groups, with no notable differences in mOS and 3‐year OS rates. However, the QHP group showed a significantly lower rate of myelosuppression compared to the LIC group (28.57% vs. 73.33%). This suggests that QHP can be considered a feasible alternative treatment option for eAML patients who have poor tolerance to LIC [80].

## 10 Ferroptosis

Ferroptosis is an iron‐dependent non‐apoptotic cell death characterized by the accumulation of intracellular lipid peroxides. It plays a critical role in various pathological conditions. In oncology, the development of ferroptosis inducers provides a promising strategy to overcome drug resistance in cancer cells. Studies have shown that certain natural compounds can exert anti‐cancer effects by modulating ferroptosis. Consequently, a deeper understanding of the molecular mechanisms governing ferroptosis and the development of agents capable of selectively inducing ferroptosis in malignant cells hold significant potential for improving cancer treatment outcomes.

AML is a refractory hematologic malignancy. Polyphyllin I (PPI), a steroidal saponin derived from the Paris genus, exhibits diverse biological activities and demonstrates significant antitumor effects against various malignancies [6]. Studies have revealed that PPI exerts notable cytotoxicity against AML MOLM‐13 cells, with an IC50 of 0.44 ± 0.09 μM. PPI induces ferroptosis by modulating the PI3K/SREBP‐1/SCD1 axis, increasing intracellular iron levels, and enhancing lipid peroxidation, showing superior efficacy compared to erastin. In vivo, administration of 4 mg/kg of PPI suppressed cancer growth, prolonged survival in mouse models, and exhibited no significant toxicity [81]. IGF2BP3, an RNA‐binding protein involved in cell growth and metabolism, regulates proliferation, differentiation, and survival by binding target mRNA. Its overexpression is strongly associated with cancer metastasis and poor prognosis. Licochalcone A (Lico A), a naturally bioactive flavonoid, has demonstrated anti‐cancer potential in multiple models. Recent studies indicate that Lico A suppresses proliferation and promotes ferroptosis in AML cells by downregulating IGF2BP3. Specifically, IGF2BP3 enhances MDM2 mRNA stability and protein expression in an m6A‐dependent manner. Lico A intervention downregulates the IGF2BP3/MDM2/p53 signaling pathway, thereby inhibiting AML cell proliferation and inducing ferroptosis. These findings provide a mechanistic basis for Lico A as a potential therapeutic agent against AML [82].

ART, a water‐soluble derivative of artemisinin, has demonstrated significant anti‐cancer activity across various malignancies. Chen et al. investigated the mechanism of ART‐induced ferroptosis in DLBCL cells through measurements of malondialdehyde and ROS. Their findings indicate that ART enhances cellular susceptibility to ferroptosis by downregulating STAT3 protein expression, effectively suppressing the malignant progression of DLBCL cells [83]. Currently, treatment strategies for BL patients over 60 years of age remain suboptimal. ART has been shown to exert inhibitory effects on BL through induction of endoplasmic reticulum stress. Studies reveal that ART activates the ATF4‐CHOP‐CHAC1 signaling pathway, thereby promoting ferroptosis in DAUDI and CA‐46 cells. In vivo experiments further confirm that ART effectively inhibits CA‐46 cells proliferation and induces apoptosis [84].

## 11 Discussion

Hematological malignancies represent a group of highly heterogeneous clonal disorders that severely compromise the hematopoietic and immune systems. Although modern therapeutic modalities—including radiotherapy, chemotherapy, immunotherapy, and targeted therapies—have improved patient outcomes to some extent, significant challenges such as treatment resistance, disease relapse, and cumulative toxicities remain. In recent years, natural medicines have gained increasing attention in the field of hematological oncology due to their multi‐target mechanisms, favorable toxicity profiles, and potential to reverse drug resistance.

As summarized in Table 1, this review systematically catalogs a variety of natural drug constituents with anti‐hematological tumor activity, including monomers, extracts, and derivatives, along with their mechanisms of action, target malignancies, and anticancer effects. These bioactive natural compounds exert therapeutic actions through diverse pathways—such as modulation of MAPK/ERK, PI3K/AKT, and Wnt/β‐catenin signaling—leading to apoptosis induction, suppression of proliferation, cell cycle arrest, and reversal of multidrug resistance. Notably, many natural compounds (e.g., artesunate, berbamine, curcumin) exhibit polypharmacological characteristics, simultaneously engaging multiple targets and pathways, thereby offering novel strategies to overcome tumor heterogeneity and adaptive resistance.

**TABLE 1:** Mechanisms of action of natural medicine in the treatment of hematological malignancies.

Active ingredient	Action mechanism	Disease	Anti‐cancer effect
Artesunate	AKT/ERK/EGFR	BL	In vitro and in vivo pathways inhibit cell proliferation
Matrine	miR‐495‐3p/miR‐543/PDK1, Wnt/β‐catenin	AML	Inhibit cell proliferation and glycolysis
Curcumin	JAK2, STAT3, Bcl‐2, P53, P27, Bax	AML	Synergistic with arsenic trioxide, induce apoptosis and inhibit proliferation
Triptolide	WSTF‐RPB1	AML	Inhibit DNA damage repair and cell proliferation
Saikosaponin D	FTO/m6A	AML	Promote gene methylation and inhibit cell proliferation
Asperuloside	RAS/MEK/ERK	CML	Promote apoptosis and differentiation, inhibit cell proliferation
Daphnoretin	STAT3	CML	Regulate gene phosphorylation and inhibit cell proliferation
Baicalin	FOXO3/CCL22	DLBCL	Increase ROS level and induce cell apoptosis
Nobiletin	PI3K/AKT	DLBCL	Regulate gene phosphorylation and apoptotic protein to induce cell apoptosis
Bufallin	Ca2+/NFATC1/cMYC	DLBCL	Apoptosis is induced by in vivo and in vitro pathways
*Celastrus orbiculatus* extract	BAX, Caspase‐3, Bcl‐2, Bcl‐xL	BL	Regulate apoptotic proteins and induce cell apoptosis
Matrine	LMP1‐c‐Myc and CaMKIIγ‐c‐Myc	NKTCL	Regulate pathway proteins and promote apoptosis process
Cistanche phenylethanoid glycosides	SIRT2‐MDM2/P300 and PI3K/AKT	NKTCL	Activate tumor suppression signals and trigger apoptotic pathways
Indirubin	HSP90AA1, PI3K/Akt	CML	Reduce mitochondrial membrane potential and promote apoptosis process
Caulis Spatholobi	Bax, Caspase‐3, Bcl‐2	CML	Regulate apoptotic proteins and induce cell apoptosis
Ferulic Acid	TLR4/NF‐κB	AML	Regulate pathways and apoptotic proteins to induce apoptosis
Resveratrol	MRP1	AML	Inhibit drug resistance gene, reverse drug resistance and induce apoptosis
Platycodin D	PI3K/AKT and MAPK/ERK	AML	Triggers the mitochondrial apoptosis pathway
Tanshinone IIA	p53, Bax/Bcl‐2, PARP and caspase‐3	AML	Regulate apoptotic proteins and oxidative stress pathways to promote the process of apoptosis
Artesunate	ROS and Bim	AML	Promote ROS production and pro‐apoptotic protein expression, and promote apoptosis process
Realgar	Bcl‐2/Bax/Cyt‐C/AIF	APL	Regulate pathways and apoptotic proteins to induce apoptosis
Cinobufagin	β‐catenin	APL	Regulate pathways and apoptotic proteins to induce apoptosis
20(S)‐Ginsenoside Rh2	Akt/TNF‐α/caspase	APL	ROS production and mitochondrial damage were induced, and apoptosis was induced
Honokiol	mTOR/MAPK	APL	Promote endoplasmic reticulum stress and induce apoptosis
Palmitic acid	miR‐429/DNMT3A	DLBCL	Reduce gene methylation and promote autophagy
Dihydroartemisinin	Oxidative stress pathway	AML	Enhance intracellular autophagy flux and promote the activation of autophagy process
Acetylshikonin	LKB1/AMPK and PI3K/Akt/mTOR	AML	Promote autophagosome formation and induce autophagy
Shikonin	PI3K/Akt/mTOR	APL	Regulate protein phosphorylation and promote autophagy process
Hinokiflavone	JNK/p38 MAPK/NF‐κB	CML	Regulate related proteins and induce autophagy
Dihydroartemisinin	JAK2/STAT3	DLBCL	Inhibition of stem cell properties, inhibition of invasion and migration
Ginsenoside Rk3	miR‐3677‐5p/CXCL12	AML	Inhibit extramedullary infiltration pathway, inhibit cell migration and invasion
Ciwujianoside E	PI3K/AKT/TGF‐β1	BL	Inhibit glycolytic enzyme and inhibit EMT process
Polyphyllin VII	cyclin D1, CDK4, CDK6 and survivin	DLBCL	Regulate related proteins and induce cycle arrest
Dihydrocelastrol	BCR/AP‐1/junb	DLBCL	Synergistic doxorubicin induces cycle arrest
Matrine	CaMKIIγ/c‐Myc/CDK6	DLBCL	Shorten the half‐life of key proteins and induce cycle arrest
Myricetin	BTK/ERK/AKT and cyclinB1/D1	DLBCL	Inhibit protein phosphorylation and block pathway transduction, induce cycle arrest
Artesunate	NF‐κB/AP‐1	PEL	Inhibit related genes, increase ROS production and DNA damage, and induce cycle arrest
Ginsenoside Rd	ERK/GSK‐3β	PEL	Regulate pathways and related proteins to induce cycle arrest
Hinokiflavone	MAPK/NF‐κB	CML	Regulate pathways and related proteins to induce cycle arrest
Sinomenine	Mitochondrial pathway	CML	Regulate mitochondrial pathway and induce cycle arrest
Polyphyllin VII	Mitochondrial pathway	CML	Regulate mitochondrial pathway and induce cycle arrest
Dihydroartemisinin	Mitochondrial apoptosis pathway	PEL	Synergistically enhance adriamycin‐induced apoptosis and tumor progression in vivo and in vitro
β‐Sitosterol	ASM/ceramide	DLBCL	Regulating sphingolipid metabolism, enhancing the efficacy of rituximab and reversing drug resistance
Artesunate	STAT3	DLBCL	Sorafenib induced apoptosis and iron death were enhanced
Matrine	Mitochondrial apoptosis pathway	CTCL	The apoptosis induced by Chidamide was enhanced
Homoharringtonine	c‐MYC/DDIT3/PUMA	AML	Activate the comprehensive stress response and synergistically enhance Azacitidine induced apoptosis
Ginsenoside K	Mitochondrial pathway	AML	Synergistically enhance DNA damage and chemotherapy resistance induced by Ara‐C
Resveratrol	The ABC transporter pathway	AML	Down‐regulated drug‐resistant protein expression and reversed MDR
Homoharringtonine	Mitochondrial pathway	AML	Decreased mitochondrial membrane potential and enhanced apoptosis induced by giltinib
Berbamine	p‐BTK/p‐AKT/CREB and GSK3β/BCL‐XL	AML	Synergistically enhance the apoptosis process induced by ibrutinib
Andrographolide	PI3K/AKT/mTOR	ALL	Regulate autophagy related genes and pathways to synergically enhance the efficacy of dexamethasone
FA‐2‐b‐β	Wnt/β‐catenin	ALL	Inhibit drug resistance genes, reduce mitochondrial membrane potential and reverse cell drug resistance
20(S)‐Ginsenoside Rh2	Lactic acid metabolic pathway	APL	Inhibition of lactate modification gene and improvement of drug resistance pathway
Baicalein	JAK2/STAT5	CML	Induces gene demethylation and reverses imatinib resistance
Myricetin	eIF4E/Ak	CML	The expression of phosphorylated protein was decreased and the progression of imatinib resistance was inhibited
Icaritin	Jak2/Stat3/p21	CML	Increased ROS accumulation and induced imatinib‐resistant cells to senescence
Homoharringtonine	Oxidative phosphorylation pathway	CML	Destroy OXPHOS and reduce ATP, inhibit the proliferation of drug‐resistant cells
Celastrol	DNA damage pathway	CML	Promote DNA damage and induce apoptosis of drug‐resistant cells
Realgar	T cell pathway	Lymphoma	Construct nanoparticles to enhance T cell toxicity
Curcumin	XIAP, Mcl‐1, IL‐6 and TNF‐α	HL	Nanoparticles were constructed to enhance the efficacy of bleomycin and doxorubicin
Realgar	BCR‐ABL1 and PTK	CML	Nanoparticles were constructed to synergically enhance imatinib effect and reverse drug resistance
Artesunate	Apoptosis pathway	DLBCL	DNA insertion agent was constructed to induce cell apoptosis
Sijunzi decoction	/	DLBCL	No disease progression or adverse drug reaction was observed after continuous use of the drug
Realgar‐indigo naturalis formula	ACHE, NCOA2, RXRA	APL	RIF combined with ATRA can promote cell apoptosis
Homoharringtonine	/	AML	The efficacy and safety of HTT are superior to etoposide
Qinghuang Powder	/	AML	QHP is equivalent to LIC but more secure than LIC
Polyphyllin I	PI3K/SREBP‐1/SCD1	AML	Increase intracellular iron concentration and lipid peroxidation, and induce iron death
Licochalcone A	IGF2BP3/MDM2/p53	AML	Down‐regulated the expression of key proteins and promoted iron death in cells
Artesunate	STAT3	DLBCL	STAT3 was down‐regulated, and the sensitivity of cells to iron death was increased
Artesunate	ATF4‐CHOP‐CHAC1	BL	Induce endoplasmic reticulum stress and promote cell iron death

Of particular importance is the observed synergistic effect when natural medicines are combined with conventional chemotherapeutic or targeted agents. Data presented in Table 1 demonstrate that various natural components can enhance the sensitivity of traditional drugs and counteract resistance mechanisms by targeting key nodes such as STAT3, BCL‐2, and MRP1. Advances in drug delivery systems (e.g., nanoparticle‐based platforms) have further improved the bioavailability and targeting efficiency of natural medicines, facilitating their clinical translation.

Nevertheless, several challenges impede progress in this field. First, the chemical complexity of natural medicines complicates the identification, standardization, and quality control of active ingredients—a crucial hurdle for clinical application. Although many promising compounds are listed in Table 1, key pharmacokinetic parameters and effective plasma concentrations remain undetermined for most. Second, despite abundant preclinical evidence, large‐scale, multicenter randomized controlled trials are scarce, leaving clinical efficacy and safety incompletely validated. Moreover, pharmacokinetic/pharmacodynamic interactions between natural and conventional therapies are poorly understood, and safety in special populations requires further evaluation.

## 12 Conclusion

This review provides a comprehensive overview of the potential applications and mechanisms of natural medicines in the treatment of hematological malignancies. Our findings indicate that multiple natural bioactive compounds significantly inhibit malignant progression by modulating various signaling pathways. As detailed in Table 1, these compounds and their mechanistic profiles serve as a valuable resource for future research. Importantly, natural medicines demonstrate substantial synergistic effects and an ability to reverse drug resistance when used in combination with conventional therapies.

To address existing challenges and leverage these promising findings, future research should focus on the following priorities: establishing robust standardization and quality control protocols for natural active ingredients; elucidating the mechanistic basis of natural product synergism with conventional treatments; promoting well‐designed multicenter clinical trials, particularly in patients with refractory or relapsed disease; and developing novel drug delivery systems to enhance targeting and bioavailability. Through interdisciplinary collaboration, natural medicines are poised to play an increasingly important role in the precision treatment of hematological malignancies, offering new strategies to improve patient prognosis and quality of life.

Natural medications have demonstrated considerable potential in the development of therapeutic agents against hematological malignancy. They complement existing treatments by enhancing efficacy, reversing drug resistance, and improving patient prognosis and quality of life. In the future, with a deeper understanding of natural medications and more precise clinical applications, they will play a more critical role in the field of hematological malignancy treatment, providing better, safer, and more effective therapeutic regimens and medication options for clinical management of hematological malignancies.

## Author Contributions

**Yaoyao Tian:** writing – original draft. **Baoyi Ni:** writing – original draft. **Dandan Cai:** writing – review and editing. **Jia Wang:** resources, writing – review and editing. **Jilai Zhou:** data curation, investigation, writing – review and editing. **Mingqian Song:** writing – review and editing. **Xi Zhang:** writing – review and editing. **Jiakang Jiang:** conceptualization, methodology, writing – review and editing.

## Conflicts of Interest

The authors declare no conflicts of interest.

## Data Availability

The authors have nothing to report.
